# Construction and evaluation of multisite recombinatorial (Gateway) cloning vectors for Gram-positive bacteria

**DOI:** 10.1186/1471-2199-8-80

**Published:** 2007-09-19

**Authors:** Tania M Perehinec, Saara NA Qazi, Sanyasi R Gaddipati, Vyvyan Salisbury, Catherine ED Rees, Philip J Hill

**Affiliations:** 1 School of Biosciences, University of Nottingham, Sutton Bonington Campus, Loughborough, Leics LE12 5RD, UK; 2 Institute of Infection, Immunity and Inflammation, Centre for Biomolecular Sciences, University of Nottingham, Nottingham NG7 2RD, UK; 3 Faculty of Applied Sciences, University of the West of England, Bristol, UK

## Abstract

**Background:**

The Gateway recombinatorial cloning system allows easy and rapid joining of DNA fragments. Here we report the construction and evaluation of three different Gram-positive vectors that can be used with the Multisite Gateway cloning system to rapidly produce new gene arrangements in plasmid constructs for use in a variety of Gram-positive bacteria.

**Results:**

Comparison of patterns of reporter gene expression with conventionally constructed clones show that the presence of residual recombination (att) sites does not have an effect on patterns of gene expression, although overall levels of gene expression may vary. Rapid construction of these new vectors allowed vector/gene combinations to be optimized following evaluation of plasmid constructs in different bacterial cells and demonstrates the benefits of plasmid construction using Gateway cloning.

**Conclusion:**

The residual *att *sites present after Gateway cloning did not affect patterns of promoter induction in Gram-positive bacteria and there was no evidence of differences in mRNA stability of transcripts. However overall levels of gene expression may be reduced, possibly due to some post-transcriptional event. The new vectors described here allow faster, more efficient cloning in range of Gram-positive bacteria.

## Background

The study of gene expression in bacteria has advanced rapidly over the last two decades. In addition to traditional mutagenesis approaches, the characterization of gene function increasingly requires the investigation of DNA segments containing promoters and their associated regulatory sequences. While many cloning and expression vectors have been developed for use in Gram-negative bacteria, there is a paucity of equivalent materials for use when working in Gram-positive bacteria.

Recombinatorial cloning systems, such as Invitrogen's 'Gateway' provide an alternative to conventional cloning that uses restriction enzymes and ligation. The Gateway system uses directed recombination between modified attachment (*att*) sites derived from *E. coli *bacteriophage λ. The λ integration system mediates two recombination reactions to promote either integration or excision of the phage genome from the *E. coli *chromosome. During integration the phage *attP *site recombines with the related, but non-identical, bacterial *attB *site producing hybrid *attL *and *attR *sequences located to the left and right of the phage genome, respectively. This recombination is mediated by integrase (Int) and integration host factor (IHF). To achieve excision of the phage from the chromosome *attL*and *attR *are recombined to regenerate *attB *and *attP *in a reaction mediated by Int in combination with the excisionase (Xis) and IHF [see [1]]. For the Gateway system, modified *att *recombination sites have been developed with recombination specificity, such that the variant *attB1 *will recombine with *attP1 *but not with other *attP *variants. Introducing these variant *att *sites at the ends of fragments to be cloned allows them to be recombined with vectors containing cognate *attB *sites, maintaining the orientation of DNA fragments during the *in vitro *recombination [2-4]. To facilitate the recombinatorial cloning, the appropriate enzymes are supplied by the manufacturer as BP clonase, that mediates *attB*/*attP *recombination events, and LR clonase to mediate recombination between *attL*/*attR *sequences.

**Figure 1:**
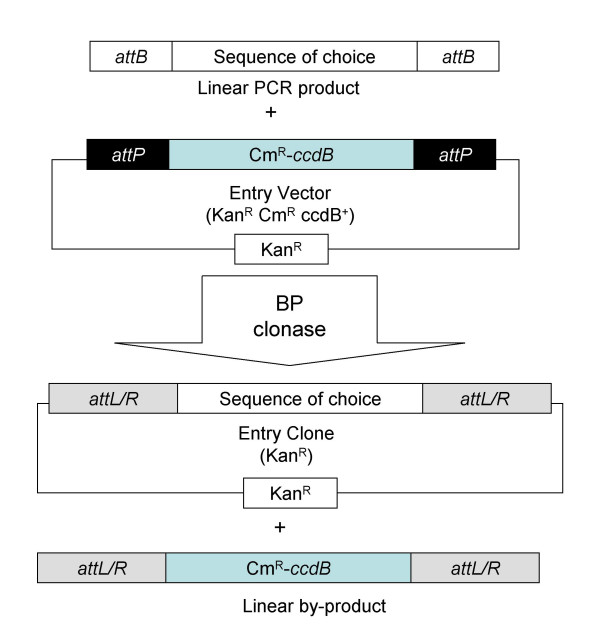
**Construction of Entry Clones using the BP reaction**. The BP recombination transfers a DNA fragment flanked by *attB *sites (white boxes) into a plasmid containing cognate *attP *sites (black boxes), termed an Entry Vector. The resultant Entry Clone contains the desired DNA fragment flanked by *attR *or *attL *sites (grey boxes), depending on the orientation of the *attB *and *attP *sites.

**Figure 2:**
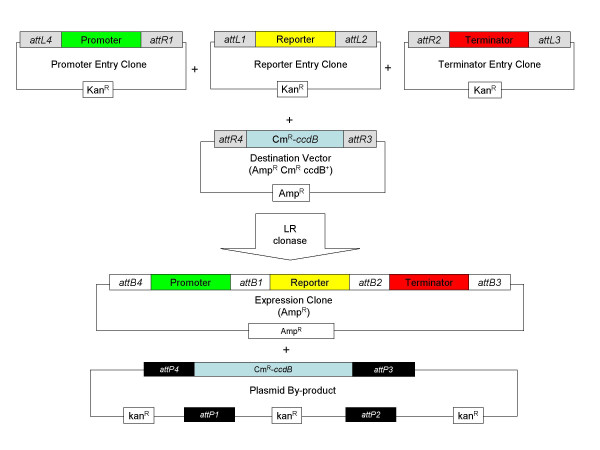
**Construction of Multisite Expression Clones using LR reaction**. Three Entry Clones with the DNA fragments of interest flanked by *attL4 *and *attR1 *(Promoter), *attL1 *and *attL2 *(Reporter gene), and *attR2 *and *attL3 *(transcriptional terminator) recombine with a Destination Vector containing *attR4 *and *attR3*. During the LR reaction *attL *sites react only with their cognate *attR *sites resulting in a Destination Clone containing all three fragments fused together into the desired expression cassette. Expression Clone constructs are selected on the basis of the antibiotic resistance gene in the Destination vector. The by-product of this reaction is non-replicative and its loss from the cell is further ensured by the presence of the *ccdB *counter-selectable marker gene.

**Figure 3:**
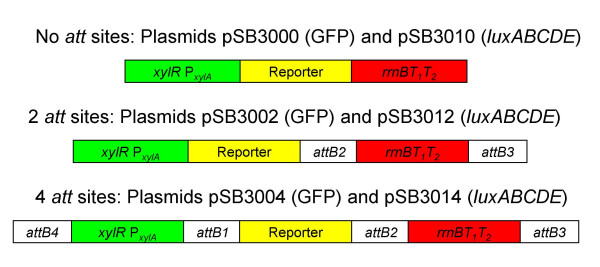
**Structure of Expression Cassettes**. Schematic of the different xylose-controllable expression cassettes created in pMK4, showing the plasmid name, corresponding reporter gene present and location of *att *sites for each construct.

The Multisite Gateway cloning technology is designed to place three DNA fragments adjacent to each other in a specific order and orientation. The procedure is carried out in two stages, the first of which is a BP reaction to transfer a linear DNA fragment flanked by *attB *sites (generated by PCR) into a plasmid, termed an Entry Vector, containing cognate *attP *sites. This creates Entry Clones containing the desired DNA fragment flanked by *attR *or *attL *sites. These can be selected by loss of the counter-selectable marker [*ccdB*; [5]] present in the original Entry Vector after transformation into *E. coli *(see Fig. 1). To create three fragment gene fusions, three such Entry Clones containing different *attL/attR *variants are created and then utilised in a second recombination step with a Destination Vector containing appropriate *attR *sites and *ccdB *using LR clonase (Fig. 2). Selection of correct Destination Clones is again facilitated by counter selection of *ccdB *that should be lost from the Destination Vector during the recombination event. The major advantage of this system is its ease and efficiency but residual *att *sites are left flanking DNA fragments (Fig. 3) and there has been some concern that these may affect gene expression from the plasmid constructs created.

The Gateway system has been primarily developed for Gram-negative bacteria or eukaryotic systems. Recently Bae and Schneewind [6] have described the development of simple Gateway vectors for use in Gram-positive bacteria, where only one fragment at a time is recombined into a Destination vector. We present here the construction of Multisite Gateway Destination Vectors based upon 3 different Gram-positive plasmid replicons that will allow the use of this rapid cloning technique in a wide range of Gram-positive bacterial hosts. These were used to construct reporter gene plasmids with the general structure Promoter:Reporter:Terminator which were then used to introduce both *lux *and *gfp *reporter genes into *Staphylococcus aureus, Listeria monocytogenes *and *Bacillus subtilis*. To determine whether the residual *att *sites have any effect on gene expression, reporter gene expression from a Promoter:Reporter:Terminator cassette constructed using either Gateway or conventional cloning methods were compared.

## Results

### Constructing a Gram-positive destination vector

A prerequisite for the Multisite Gateway system is a Destination vector containing a Cm^R^-*ccdB *cassette flanked by *attR4 *and *attR3 *sites. This was amplified from pDESTR4-R3 (Invitrogen) using primers PmeattR3 and PmeattR4 (Table 1) to generate a 1770 bp PCR product. This amplicon was cut with *Pme*I and ligated into the Gram-positive shuttle vectors pMK4 (*Sma*I site), pHB201 (*Pvu*II/*Pme*I sites) and pUNK1 (*Sma*I site). These recombinant plasmids were used to transform *E. coli *DB3.1 (*gyrA*) that can tolerate the *ccdB *gene. Resulting plasmids were characterised by restriction mapping and sequence analysis. These new Gram-positive destination vectors were given the designation pDEST-pMK4, pDEST-pHB201 and pDEST-pUNK1, respectively.

**Table 1:** Primers for PCR and sequencing

Gateway primers	Sequence (5-3')	Use
Term attB2F	GGGGACAGCTTTCTTGTACAAAGTGGAAATCAGAACGCAGAAGCGGTC	Amplification of terminator
Term attB3R	GGGGACAACTTTGTATAATAAGTTGTTTAAACTGGCAGTTTATGGCGGGC	Amplification of terminator
Lux E attB2R	GGGGACCACTTTGTACAAGAAAGCTGGGTTCAACTATCAAACGCTTCGG	Amplification of *lux *reporter
GFP attB2R	GGGGACCACTTTGTACAAGAAAGCTGGGTGACACATTTATTTGTATAGTTC	Amplification of *gfp *reporter
GFPopt attB1F	GGGGACAAGTTTGTACAAAAAAGCAGGCTAGGAGGAATAAAAAATGAGTAAAGGCGAAGAAC	Amplification of *gfp *reporter
XylRA attB4F	GGGGACAACTTTGTATAGAAAAGTTGCGTTGACTTAACTAACTTATAG	Amplification of *xylR *P_*xylA*_
XylRA attB1R	GGGGACTGCTTTTTTGTACAAACTTGTTGA TTTAAGTGAACAAGTTTATC	Amplification of *xylR *P_*xylA*_
XylA attB4F	GGGGACAACTTTGTATAGAAAAGTTGAAACATTGAAATAAACATTTATTTTGTATATGATG	Amplification of P_*xylA*_
XylA attB1R	GGGGACTGCTTTTTTGTACAAACTTGTTGATTTAAGTGAACAAGTTTATC	Amplification of P_*xylA*_
LuxA attB1F	GGGGACAAGTTTGTACAAAAAAGCAGGCTAGGAGGACTCTCTATGAAATTTGGAAAC	Amplification of *lux *reporter
SASP attB4F	GGG GAC AAC TTT GTA TAG AAA AGT TGT TTA AAC GCT GGA ACC TTT TGT TCC CAA AAG	Amplification of P_*sspA*_
SASP attB1R	GGG GAC TGC TTT TTT GTA CAA ACT TGT TTT ATT TAG TAT GGT TGG GTT AAC TGG	Amplification of P_*sspA*_
attR3	GGGGCGTTTAAACAAGCTTGTAAAACGACGGCCAGTG	sequencing
PMEattR4	GGGTTTAAACAACTTTGTATAGAAAAGTTGAACGAG	sequencing
Ccdb Rev	AGGAAGGGATGGCTGAGGTC	sequencing
Cm R4-R3F	CAGGCGGGCAAGAATGTGA	sequencing

### Entry clone construction

A number of Entry Clones using the Entry Vectors from the Gateway construction kit were then prepared to allow us to rapidly create reporter plasmids for use in Gram-positive bacteria. In each case the required sequence was amplified by PCR using the primers listed in Table 1 and introduced into the entry vectors using the BP recombination reaction (see Fig. 2) and each of the resulting Entry Clones was characterised by restriction mapping and sequence analysis. First a 3' Entry Clone was created using plasmid pDONOR-P2R-P3 containing the transcriptional terminator (*rrnBT1T2*) amplified from pTRC99A as *E. coli *terminators have been previously demonstrated to function in Gram-positive bacteria [7]. Secondly a series of reporter gene entry clones were created using pDONOR-221. These contained either the *gfp *or *luxABCDE*, or both reporter systems (*gfp*:*luxABCDE*) [8-10]. Finally a series of 5' entry clones were created using plasmid pDONOR-P4-P1R. The promoter fragments chosen were those that we have previously used to create reporter gene constructs in Gram positive bacteria and included xylose utilisation gene (P_*xylA *_[11]) amplified from *B. megaterium *chromosomal DNA and the small acid soluble protein (SASP) gene promoter (P_*sspA*_: [12]) amplified from *B. subtilis *chromosomal DNA. In addition P_*xylA *_was also amplified from *B. megaterium *along with its cognate regulator protein XylR (*XylR *P_*xylA *_[11]).

### Construction of reporter plasmids using Gateway recombination

After generating the Entry Clones containing the promoters, reporter genes and transcriptional terminator, the MultiSite Gateway LR reaction was performed with the new Gram-positive destination vectors to create expression clones with the following structure, *attB4*-promoter-*attB1*-reporter gene-*attB2*-terminator-*attB3 *(see Fig. 2). Each LR reaction mixture was transformed into *E. coli *Top10 competent cells to select for loss of the *ccdB *gene. Transformants were then screened for the expression of the appropriate reporter genes and in each case the structure of clones was confirmed by restriction and sequence analysis. All final expression constructs created using this Gateway system contain 4 *attB *sites (see Fig. 2).

### Construction of expression clones for *Staphylococcus aureus*

Previously we have successfully used reporter gene constructs based on the shuttle vector pMK4 for studies in Gram-positive bacteria [8-10], therefore pDEST-pMK4 was chosen as the first destination vector to be evaluated. Constructs were created containing *xylR *P_*xylA *_fused to either the *gfp *or *luxABCDE *followed by the terminator sequence (pSB3004 and pSB3014, respectively; see Fig. 3 and Table 2) and these were transformed into *S. aureus *RN4220.

**Table 2:** Plasmids used in study

Plasmid	Features of plasmid	Reference/source
pTMP100	pMK4 containing *S. aureus *P_*rpsJ*_:*gfp*:*luxABCDE*	Hill laboratory collection
pTRC99A	*rrnB *T1, *rrnB *T2	27
pDONRP4-P1R	Entry vector to clone *attB4 *and *attB1 *flanked PCR products, Km^r^, Cm^r^, *ccdB*^+^	Invitrogen
pDONR221	Entry vector to clone *attB1 *and *attB2 *flanked PCR products, Km^r^, Cm^r^, *ccdB*^+^	Invitrogen
pDONR P2R-P3	Entry vector to clone *attB2 *and *attB3 *flanked PCR products, Km^r^, Cm^r^, *ccdB*^+^	Invitrogen
pDESTR4-R3	Destination vector, contains *attR4/R3 *sites. Recombines with entry clones in a Multisite Gateway LR reaction, Ap^r^, Cm^r^, *ccdB*^+^	Invitrogen
pHB201	*B. subtilis ori*-pBR322, *ori*-1060, *cat*86:*lacZα *Cm^r ^Em^r^	15
pMK4	Gram-Positive Shuttle vector, Ap^R^, Cm^r^	28
pSB2018	pMK4 containing *xylR *P_*xylA*_:*gfp*	8
pSB2026	pMK4 containing *xylR *P_*xylA*_:*luxABCDE*	Hill laboratory collection
pUNK1	Gram-positive shuttle vector, Em^r^	14
pDEST-pMK4	pMK4 containing *attR4 *and *attR3 *sites for recombination with entry clones in an LR reaction. Ap^r^, Cm^r^, *ccdB*^+^	This work
pDEST-pUNK1	pUNK1 containing *attR4 *and *attR3 *sites for recombination with entry clones in an LR reaction. Em^r^, Cm^r^, *ccdB*^+^	This work
pDEST-pHB201	pHB201 containing *attR4 *and *attR3 *sites for recombination with entry clones in an LR reaction. Em^r^, Cm^r^, *ccdB*^+^	This work
pSB3000	3 fragment expression cassette in pMK4. *xylR *P_*xylA*_:*gfp*:*rrnBT1T2 *(0 *attB*)	This work
pSB3002	3 fragment expression cassette in pMK4. *xylR *P_*xylA*_:*gfp*:*rrnBT1T2 *(2 *attB*)	This work
pSB3004	3 fragment expression cassette in pMK4. *xylR *P_*xylA*_:*gfp*:*rrnBT1T2 *(4 *attB*)	This work
pSB3005	3 fragment expression cassette in MK4. P_*xylA*_:*gfp*:*luxABCDE rrnBT1T2 *(4 *attB*)	This work
pSB3007	3 fragment expression cassette in pUNK1. P_*xylA*_:*gfp*:*luxABCDE*:*rrnBT1T2 *(4 *attB*)	This work
pSB3010	3 fragment expression cassette in pMK4. *xylR *P_*xylA*_:*luxABCDE*:*rrnBT1T2 *(0 *attB*)	This work
pSB3012	3 fragment expression cassette in pMK4. *xylR *P_*xylA*_:*luxABCDE*:*rrnBT1T2 *(2 *attB*)	This work
pSB3014	3 fragment expression cassette in pMK4. *xylR *P_*xylA*_:*luxABCDE*:*rrnBT1T2 *(4 *attB*)	This work
pSB3024	3 fragment expression cassette in pHB201. P_*sspA*_*gfp*:*luxABCDE*:*rrnBT1T2 *(4 *attB*)	This work

To investigate whether the presence of the *attB *sites between the various elements of the expression cassettes affected patterns of gene expression, a series of parallel constructs in pMK4 were created by conventional cloning. To remove the *att *sites flanking the promoter in pSB3004, the promoter and *gfp *reporter gene were replaced with an equivalent fragment from pSB2018 [8] that contains no *att *sites (see methods section for details of plasmid construction). Similarly to remove the *attB *sites flanking the promoter in the *luxABCDE *expression vector pSB3014, the '*bla xylR *P_*xylA*_*luxAB *fragment was replaced with an equivalent fragment from pSB2026 [9] which again contains no *attB *sites. The resulting *gfp *and *lux *constructs were designated pSB3002 and pSB3012, respectively. These constructs have two *attB *sites remaining, namely *attB2 *and *attB3 *flanking the terminator (Fig. 3).

To construct a plasmid with an equivalent structure to pSB3004 but without any *attB *sites, a terminator sequence was inserted into plasmid pSB2018 in a *Pst*I site located immediately downstream of the P_*xylA*_*gfp *sequence. The resulting plasmid was designated pSB3000 and is equivalent to pSB3002 but contains no *attB *sites flanking the terminator (Fig. 3). To create the equivalent *luxABCDE *reporter plasmid with no *attB *sites, the *gfp *gene was excised from pSB2018 and a *luxABCDE *fragment inserted in its place. The resulting plasmid was digested with *Pst*I and the *rrnBT1T2 *fragment cloned downstream of the *lux *operon. This clone was designated pSB3010 (Fig. 3) and sequencing analysis was used to confirm the presence of the transcriptional terminator, promoter and reporter genes in all new reporter constructs and successful deletion of *attB *sites.

These experiments created pMK4-based plasmids where no *attB *sites were present (pSB3000 and pSB3010) or with only two *attB *sites flanking the terminator sequence (pSB3002 and pSB3012) or where 4 *attB *sites were present (pSB3004 and pSB3014) (see Table 2 and Fig. 3). All of these plasmids contained the reporter genes under the control of the xylose inducible promoter, P_*xylA*_, and were transformed into *S. aureus *RN4420.

### Evaluation of expression clones in *Staphylococcus aureus*

**Figure 4:**
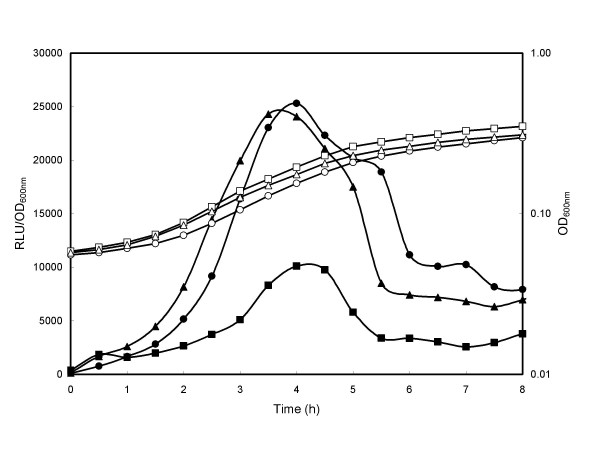
**Effect of *att *sites on reporter gene expression in *S. aureus***. *S. aureus *RN4220 with *lux *expression plasmids pSB3010 (○, ●; 0 *att *sites), 2 pSB3012 (△, ▲ 2 *att *sites) or pSB3014 (□, ■; 4 *att *sites) were grown in LB medium containing 0.5% (w/v) xylose at 37°C. Growth (OD 600 nm; open symbols) and luminescence (Relative Light Units; RLU closed symbols) were monitored over time. Reporter gene data are presented as RLU/OD_600 nm _to account for increasing cell number during the experiment.

First the growth and expression of the P_*xylA *_*lux *expression plasmids (pSB3010 [no *attB *sites], pSB3012 [[2]*attB *sites] and pSB3014 [[4]*attB *sites]) were compared in rich broth when the promoter systems were fully induced by addition of xylose throughout growth (Fig. 4). No significant differences were seen in the growth of bacteria harboring analogous plasmids and, as previously reported [9], expression of the *lux *operon from this promoter was seen to reach a maximum during the logarithmic growth phase. Bioluminescence from expression plasmids pSB3010 and pSB3012 was approximately equivalent however light emission from pSB3014, which has two additional *attB *sites flanking the promoter region, consistently showed a lower light level, with typically half the bioluminescence intensity of the other two plasmid constructs tested. Similar differences in light level from the 4 *att *constructs were also noted when *lux *expression from the plasmids in *L. monocytogenes *was compared (data not shown).

**Figure 5:**
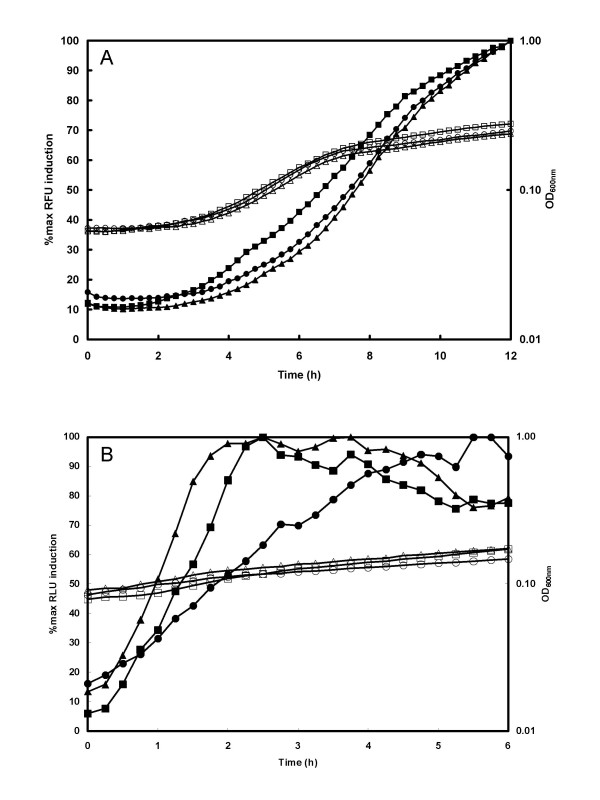
**Reporter gene induction kinetics**. *S. aureus *RN4220 containing reporter plasmids pSB3004 (□, ■; 4 *att *sites), pSB3002 (△, ▲; 2 *att *sites) and pSB3000 (○, ●; no *att *sites) were grown to mid-log phase in Tris Minimal Succinate medium with 5 μg ml^-1 ^Cm and then diluted 1/20 into fresh medium supplemented with 0.5% xylose. Triplicate samples were placed in a 96 well microtitre plate and incubated at 37°C in a Tecan Genios Pro. Panel A: fluorescence (solid symbols, Relative Fluorescence Units; RFU) and absorbance (open symbols) were measured at 10 min intervals. Plasmids used were pSB3004 (□, ■; 4 *att *sites), pSB3002 (□, ▲; 2 *att *sites) and pSB3000 (○, ●; no *att *sites). Panel B. luminescence (solid symbols, Relative Light Units; RLU)) and absorbance (open symbols) were measured at 10 min intervals. Plasmids used were pSB3014 (□, ■; 4 *att *sites), pSB3012 (△, ▲; 2 *att *sites) and pSB3010 (○, ●; no *att *sites). Data is presented as % maximal signal to allow direct comparison of expression kinetics despite the fact that light levels from each construct were different.

To try to elucidate whether the lower bioluminescence observed when the promoter was flanked by *attB *sites was due to differences in the rate of transcription or translation, a second series of experiments were carried out in minimal media where the reporter gene expression was only induced by addition of 0.5% (w/v) xylose once bacteria had entered the exponential phase of growth, and the rate of gene induction was then followed. Both *lux *(pSB3010, pSB3012 and pSB3014) and *gfp *(pSB3000 [no *attB *sites], pSB3002 [[2]*attB *sites] and pSB3004 [[4]*attB *sites]) expression plasmids were used for these experiments (Fig. 5). The induction of P_*xylA *_led to a rapid increase in fluorescence (Fig. 5a) and luminescence (Fig. 5b) for all reporter constructs. Sequences adjacent to ribosome binding sites have previously been shown to affect translational efficiency [13]. In our study no significant differences were found in the maximum rates of induction of reporter activity when analogous plasmids were compared. This indicates that neither the initiation of transcription nor translation were affected by *attB *sites proximal to the promoter or RBS of the reporter genes.

**Figure 6:**
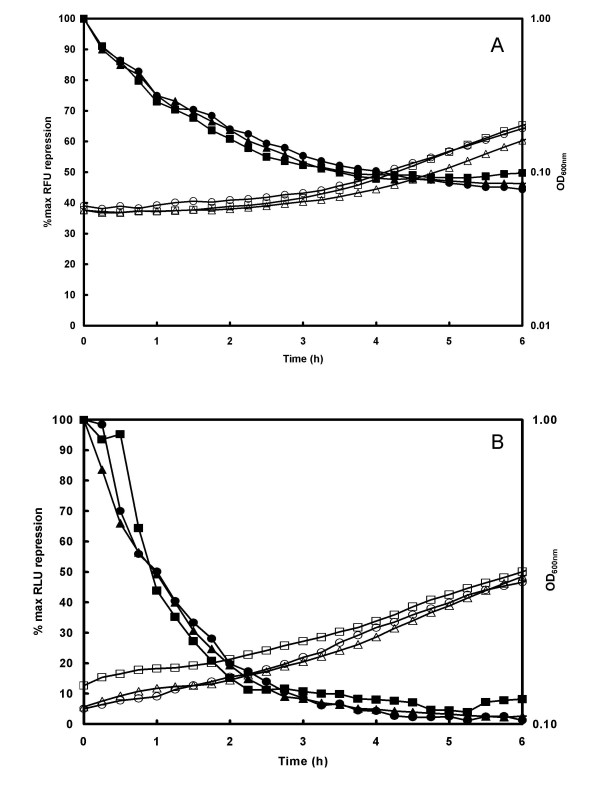
**Reporter gene repression kinetics**. *S. aureus *RN4220 containing reporter plasmids pSB3004 (□, ■; 4 *att *sites), pSB3002 (△, ▲; 2 *att *sites) and pSB3000 (○, ●; no *att *sites) were grown to mid-log phase in Tris Minimal Succinate medium with 5 μg ml^-1 ^Cm and 0.5% xylose. Bacteria were washed and resuspended in an equal volume of medium containing 5 μg ml^-1 ^Cm and 1% glucose. Aliquots were placed in a 96 well microtitre plate and incubated at 37°C in a Tecan Genios Pro. Panel A: fluorescence (solid symbols, (Relative Fluorescence Units; RFU) and absorbance (open symbols) were measured at 10 min intervals. Plasmids used were pSB3004 (□, ■; 4 *att *sites), pSB3002 (□, ▲; 2 *att *sites) and pSB3000 (○, ●; no *att *sites). Panel B: luminescence (solid symbols, Relative Light Units; RLU) and absorbance (open symbols) were measured at 10 minute intervals. Plasmids used were pSB3014 (□, ■; 4 *att *sites), pSB3012 (□, ▲; 2 *att *sites) and pSB3010 (○, ●; no *att *sites). Data is presented as % maximal signal to allow direct comparison of repression kinetics despite the fact that light levels from each construct were different.

To ascertain if the presence of *attB *sites had any effect on mRNA stability, repression of reporter gene transcription was monitored following removal of xylose from xylose-induced cultures. As shown in Figures 6a and 6b, removal of xylose from logarithmically growing cells containing the reporter plasmids resulted in an immediate reduction of signal from both *gfp *and *lux *expression plasmids. A faster decrease in signal was seen in bacteria harbouring the *lux *constructs compared to the equivalent *gfp *constructs, as would be expected due to the longer half-life of the GFP reporter molecule [9]. However the rate of loss of signal was the same for all constructs indicating that the presence of *attB *sites within the reporter gene structure did not significantly alter the t_1/2 _of the mRNA.

### Evaluation of expression clones in *Listeria monocytogenes*

**Figure 7:**
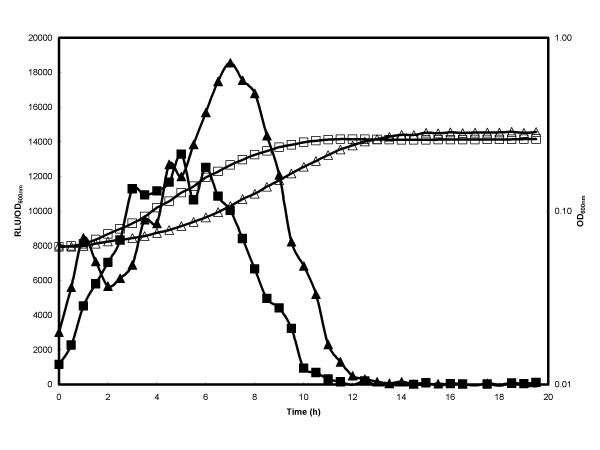
**Comparison of vectors in *L. monocytogenes***. *L. monocytogenes *7973 plasmids based on the shuttle vector pMK4 (△, ▲; pSB3005) or pUNK1 (□ ■; pSB3007) containing the *gfp:luxABCDE *operon under the control of the P_*xylA*_promoter (see Table 2). Cells were grown in MWB at 37°C and luminescence (closed symbols, Relative Light Units; RLU)) and growth measurements (open symbols) were taken at intervals. Reporter gene data are presented as RLU/OD_600 nm _to account for increasing cell number during the experiment.

Plasmid pMK4 is also known to replicate in *L. monocytogenes *[8] and in this case a dual reporter plasmid containing both *gfp *and the Gram-positive optimised *lux *operon was created for evaluation in *L. monocytogenes*. Plasmid pSB3005 contained the P_*xylA *_promoter fused to the *gfp*:*luxABCDE *dual reporter operon and the *rrnBT1T2 *transcriptional terminator in pDEST-pMK4. When *L. monocytogenes *NCTC 7973 cells containing pSB3005 were grown in the presence of selective antibiotics, cultures showed an extended lag phase but eventually grew at normal rates and achieved a cell density expected for *L. monocytogenes *cultures (see Fig. 7). When *L. monocytogenes*(pSB3005) cells were visualised by fluorescence microscopy after overnight growth with antibiotic selection, only approx. 20% of the cells expressed GFP, suggesting either a high rate of selection of non-expressing clones or plasmid instability.

While pMK4 has been found to be stable in *S. aureus *[9,10], some plasmid instability had been observed in *L. monocytogenes *(Hill, unpublished data) therefore plasmid stability experiments were performed. To rule out any effect on plasmid stability caused by expression of the dual reporter gene operon, *L. monocytogenes *7973(pMK4) cells were also examined. After overnight culture (15 generations) without antibiotic the average percentage loss of pMK4 plasmids was 95% demonstrating that the plasmids were not stably maintained in *L. monocytogenes *without selection. Individual values for plasmid loss with or without the reporter gene were 94% (pMK4) or 97% (pSB3005), respectively, and these values were not significantly different (P = 0.05). Therefore instability could not be attributed to expression of the dual reporter gene operon. When bacteria were grown in the presence of Cm (7 μgml^-1^) selection, average plasmid loss for both plasmids was still high but decreased to 73%. This was a surprising result since the presence of the antibiotic was expected to completely stabilise the plasmid. Again no significant difference in values for reporter plasmid and parent vector were found.

To address this problem, a new reporter gene construct (pSB3007) was created in the vector pDEST-pUNK1 [14] containing P_*xylA*_:*gfp*:*luxABCDE*:*rrnBT1T2*. Both pSB3007 and pUNK1 were transformed into *L. monocytogenes *7973 and plasmid stability experiments carried out. Average plasmid loss of pUNK1 plasmids from cells grown in the absence of antibiotic was only 25% (pUNK1) and 12% (pSB3007). For bacteria grown in the presence of Erm (5 μgml^-1^) average plasmid loss did not improve and was similar for pUNK1 and pSB3007 at approx. 22%. This demonstrated that the pUNK1 plasmids are intrinsically more stable in *L. monocytogenes *cells than the pMK4-based constructs and shows that expression of the reporter genes does not lead to higher levels of plasmid loss. Little additional plasmid stability was achieved for the pUNK1 plasmids when antibiotic selection was applied, showing that these plasmids are appropriate to use in circumstances where antibiotics cannot be used for plasmid selection.

To investigate whether the expression of the dual reporter operon was stable, microscopic examination of bacteria containing pSB3007 grown in the presence of Erm was carried out. Very few cells were non-fluorescent, confirming that the majority of plasmid-containing cells also expressed the reporter genes. Expression of the *lux *genes was evaluated during growth of *L. monocytogenes*(pSB3007) in BHI broth in the presence of Erm. In this case the extended lag phase identified when growing the pMK4-based plasmid did not occur (see Fig. 7). Bioluminescence levels increased as cells entered the exponential phase of growth and began to decline in early stationary phase (Fig. 7), following the pattern of expression previously reported for this promoter in *L. monocytogenes *[8]. This again confirmed that the presence of the *att *sites in these constructs does not change the pattern of expression of the reporter gene.

### Evaluation of *sspA *expression clones in *Bacillus subtilis*

**Figure 8:**
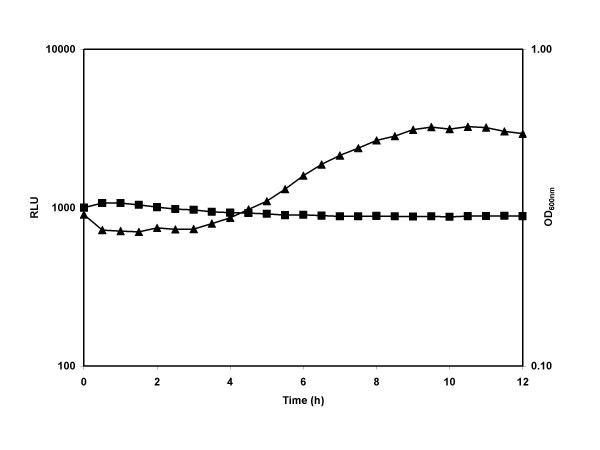
**Pattern of P_*sspA *_promoter induction in *Bacillus***. *B. subtilis*(pSB3024) containing the Gram-positive optimised dual *gfp:luxABCDE *operon under the control of P_*sspA *_(see Table 2) was grown overnight in LB supplemented with 5 μg ml^-1 ^Erm at 37°C. Cells were then harvested by centrifugation and washed with PBS before diluting 1/50 into SMM supplemented with 5 μg ml^-1 ^Erm. Cultures were then incubated at 37°C and luminescence (▲, Relative Light Units; RLU)) and absorbance measurements (■) were taken at intervals.

The differences in pMK4 plasmid stability seen in different hosts demonstrated the importance of vector choice when working in different Gram-positive bacteria. Therefore to develop a Gateway Destination vector for Bacilli, a plasmid containing the pTA1060 replicon known to be stable through sporulation in *B. subtilis *was chosen (pHB201; [12,15]). The Destination vector pDEST-pHB201 was used to create an Expression Clone containing the promoter from the sporulation-associated SASP gene (P_*sspA*_)fused to the dual reporter operon *gfp:luxABCDE *(pSB3024; see Table 2). SASP is synthesised only during sporulation to protect DNA within the spore [16]. The plasmid was transformed into the *pyrB B. subtilis *strain, BGSC 1A393, that does not grow in minimal media without added uracil. Cells were grown in rich broth and then transferred into a sporulation minimal media (SMM) without uracil supplement to encourage initiation of sporulation due to nutrient limitation [17]. As expected, no growth occurred in the SMM media, but a strong induction of reporter gene expression was seen after 3 h (Fig. 8). Light levels reach a maximum of 3000 RLU after 10 h and then began to decline. This peak of gene expression suggests that a large number of the cells in the population began sporulating at the same time, and corresponds with the expected σ^G^-dependent expression of P_*sspA *_in the developing forespore after sporulation initiation in *B. subtilis *[18].

## Discussion

The work presented here underlines the flexibility of the MultiSite Gateway Technology to assemble multiple DNA fragments precisely, efficiently, and directionally in a defined order and orientation, without subcloning, thereby negating the need for restriction enzymes and DNA ligases. One concern raised about this cloning system is the presence of the residual *attB *sites in the final Expression Clone constructs. Here we show that no significant differences are seen in rates of gene induction due to the presence of *att *sites within the expression cassettes, although in this model system the amount of resultant protein seems to be lower where *attB *sites are introduced proximal to the promoter and RBS. Comparison of GFP and Lux data indicated that mRNA half-life stability was not affected by differences in mRNA structure between the conventional and Gateway clones in *S. aureus*.

Another concern often expressed when using reporter genes for *in vivo *studies is that the expression of the reporter will place a metabolic burden on the bacteria and therefore alter growth behaviour. In this case we have used a dual reporter gene operon that encodes 6 genes in total and yet there was no evidence of even high level expression affecting bacterial growth. However it is clear that problems of vector maintenance may be of more significance than any metabolic burden of the reporter genes. In addition it is clear that antibiotic selection alone is not sufficient to ensure that all cells in a population retain reporter gene constructs, and that the choice of antibiotic marker gene is also an important factor when designing vectors. The high level of plasmid loss seen with pMK4 and its derivatives in the presence of Cm may be due to the fact that the *cat *gene confers resistance by inactivation of the antibiotic [19]. Hence during culture, levels of chloramphenicol in the media decrease and allow outgrowth of plasmid-free segregants which no longer possess the plasmid-borne *cat*. This may explain the long lag seen in the growth experiments where antibiotics were added to the growth media (see Fig. 7). In contrast the *ermAM *gene present on pUNK1 confers resistance by methylation of the rRNA [20] and therefore the antibiotic concentrations will not decline and maintain selection throughout the experiment. The lack of extended lag phase seen with pUNK1-based vectors supports this hypothesis. This makes the development of the Gateway cloning system even more attractive for Gram-positive bacteria, as it is relatively simple to generate new constructs in different vector when such effects are detected.

In this report we have used the Gateway system to rapidly generate Expression Clone plasmids, however the same Multisite DNA fragment fusion system could also be used in site directed mutagenesis strategies by amplifying upstream and downstream regions of a target gene and recombining these with a marker gene that will replace the target gene following *in vivo *recombination. Evaluation of Gram-positive suicide Destination vectors is currently underway and will facilitate mutational analysis of genes in Gram-positive bacteria.

## Conclusion

The residual *attB *sites resulting from Gateway cloning do not affect gene expression in Gram-positive bacteria any more than residual restriction sites resulting from conventional cloning. The rapidity and precision of recombinatorial cloning makes it the method of choice where precise juxtaposition cloning of DNA sequences is not required, and simplifies the construction of expression plasmids, where multiple ligation steps are usually needed. A range of vector systems with different origins of replication are needed when working with diverse Gram-positive cell types to ensure that data obtained is as robust as possible.

## Methods

### Bacterial strains and growth conditions

*E. coli *TOP10 (F^-^) and *E. coli *DB3.1 (F^- ^*gyr*A462) were cultivated in Luria-Bertani (LB; 5 g^-1^) broth or agar supplemented with appropriate antibiotics. *L. monocytogenes *NCTC 7973 (ATCC NO. serovar 1/2a) was grown in brain-heart infusion medium (BHI, Oxoid) or in the chemically defined medium Modified Welshimer's Broth (MWB; [21]). *Bacillus subtilis *BGSC 1A393 *pyrB *[22] was cultivated in LB broth or in Spizizens Minimal Media (SMM; 16). *S. aureus *RN4220 was grown in LB medium or in Tris Minimal Succinate medium (TMS; [23]). Antibiotics Ampicillin (Ap) Erythromycin (Erm) and Chloramphenicol (Cm) were used to select for plasmid maintenance when appropriate. Long term stocks of bacterial strains were stored at -80°C in Microbank™ porous beads.

### Amplification of DNA

PCR reactions contained 10 μM of each primer, template DNA at 0.2 ng μl^-1 ^and 2.5 u of AccuPrime *Taq *DNA Polymerase High Fidelity (Invitrogen) according to the Manufacturer's instructions. *Bacillus megaterium *NCTC 10342 DNA was used as template DNA for amplification of the *xylR *P_*xylA *_fragment, *B. subtilis *168 was used as template for amplification of P_*sspA *_and plasmid pTMP100 was used as template for amplification of reporter genes; this plasmid contains the dual reporter genes *gfp *and *luxABCDE *under the control of the *S. aureus *P_*rpsJ*_; all genes in the *lux *operon are modified to achieve enhanced translation in Gram-positive bacteria. PCR reactions were incubated at 94°C for 1 min, followed by 30 cycles of 94°C for 30 sec, 52°C for 30 sec, and 68°C for 3 min (or 7 min for 6 kb fragments), with a final extension period at 68°C for 10 min. If non-specific PCR products were formed, anneal temperatures were increased to a maximum of 60°C.

### DNA manipulation & vector construction

Plasmids were prepared using Qiagen DNA purification kits. Restriction enzyme analyses were performed using standard procedures [24]. T4 DNA ligase (Promega) was used for ligation of DNA fragments. PCR products and restriction fragments were purified from low melting point agarose gels using a Promega Wizard PCR purification Kit and DNA fragments eluted in water. For Gateway cloning, the Multisite Gateway Three-Fragment Vector Construction kits were used according to manufactures instructions (Invitrogen).

To create pSB3002 (2 *att *sites), pSB3004 was cut with *Sca*I/*Msc*I to release a fragment containing '*bla xylR *P_*xylA *_*gfp*'. This was replaced with a *Sca*I/*Msc*I fragment from pSB2018 (Table 2) containing the same promoter/reporter gene sequences created by conventional cloning and contains no *att *sites [8]. To create pSB3012 (2 *att *sites), pSB3014 was restricted with *Sca*I/*Mlu*I to excise the '*bla xylR *P_*xylA *_*luxAB *fragment and replaced with a *Sca*I/*Mlu*I from pSB2026 [9] containing the same sequences but without *attB *sites (see Fig. 3). Plasmid pSB3000 (no *att *sites) was constructed by insertion of the *rrnBT1T2 *sequence amplified from pTRC99A, using primers incorporating 5' *Pst*I and 3' *Nsi*I sites, into plasmid pSB2018 in the *Pst*I site immediately downstream of P_*xylA*_*gfp*. The amplicon was restricted with *Pst*I and *Nsi*I and ligated with pSB2018 cut with *Pst*I. To create pSB3010 (no *attB *sites), pSB2018 was restricted with *Sma*I/*Pst*I to remove *gfp *and then ligated with a *Sma*I/*Pst*I *luxABCDE *fragment excised from pTMP1 (Table 2). The resulting plasmid was digested with *Pst*I and the *Pst*I/*Nsi*I *rrnBT1T2 *fragment inserted downstream of the *lux *operon (Fig. 3).

### Bacterial transformation

*E. coli *transformation was performed using DB3.1 (for maintenance of *ccdB*^+ ^plasmids by virtue of their *gyr*A462 mutation) or TOP10 (for selection of Entry and Expression clones) frozen competent cells (Invitrogen). Cells were plated on LB agar supplemented with Cm (30 μgml^-1^), Kanamycin (Km; 30 μgml^-1^), Ap(50 μgml^-1^), or Em (150 μgml^-1^) as appropriate for the vector used.

Transformation of *L*. *monocytogenes *and *S. aureus *with plasmids were carried out by electroporation [25,26]. *L. monocytogenes *transformants were plated on BHI agar (Cm 7 μgml^-1^), *S. aureus *transformants were selected on LB agar (0.5% xylose, Cm 5 μgml^-1^). The one step natural transformation method was used to transform *B. subtilis *[16] and plated on LB agar (Cm 7 μg ml^-1^). Expression of *lux *and *gfp *reporter genes in individual colonies was detected using a Nightowl CCD camera system with integrated fluorescence excitation (Berthold Technologies).

### Plasmid stability measurements

Bacteria were inoculated into BHI broth, with or without antibiotic selection for the plasmid vector being used. After overnight culture at 37°C with aeration, samples were diluted and viable count determined using duplicate samples grown on plates with and without appropriate antibiotics. Plasmid loss was determined both after one night of culture (15 generations) and after subculture from this initial flask (23 generations). Plasmid loss was calculated by subtracting the number of antibiotic resistance colonies from the total number of colonies and expressing this value as a percentage of the total number of cells. Results presented are the mean of at least two experiments.

### Growth and gene expression measurements

*L. monocytogenes *or *B. subtilis *cultures were grown overnight in rich broth supplemented with suitable antibiotics at 37°C with shaking (200 rpm). Samples were centrifuged at 16,000 × *g *for 2 min at room temperature, washed twice with sterile phosphate buffered saline (PBS) before resuspending in an equal volume of PBS. The washed cell suspension was diluted 1/100 (*L. monocytogenes*) or 1/50 (*B. subtilis*) into either chemically defined or rich media supplemented with antibiotics as appropriate.

*S. aureus *overnight cultures were grown aerobically at 37°C in TMS supplemented with Cm (5 μg ml^-1^). These were diluted 1/50 into fresh medium and grown for 4 h at 37°C with or without 0.5% xylose. To measure induction of P_*xylA *_cultures were grown without xylose then 0.5% xylose was added after 4 h. To measure repression of the *xylR *P_*xylA *_promoter system, cultures were grown for 4 h with 0.5% xylose, then cells were harvested and washed (as above) and transferred into media supplemented with Cm 5 μgml^-1 ^and 1% glucose (for catabolite repression *via *XylR; [11]).

For all reporter gene measurements, replicate samples (200 μl) were placed into the wells of a 96-clear-bottom microtiter plate (Porvair) and incubated at 37°C in a Tecan Genesis Pro microplate reader. Optical density (600 nm), fluorescence (RFU; Excitation wavelength = 485 nm, Emission wavelength = 535 nm) and light levels (RLU) readings were taken at regular periods over the course of the experiment.

## Competing interests

The author(s) declares that there are no competing interests.

## Authors' contributions

TMP and SNAQ carried out cloning and growth/reporter studies and helped to draft the manuscript, SRG carried out cloning and growth/reporter studies, CEDR and VS participated in the design of the study and helped to draft the manuscript. PJH conceived of the study, and participated in its design and coordination and helped to draft the manuscript. All authors read and approved the final manuscript.
